# Recent advancements and comprehensive analyses of butyric acid in cardiovascular diseases

**DOI:** 10.3389/fcvm.2025.1608658

**Published:** 2025-07-28

**Authors:** Qiang Xu, Xiaomin Liu, Zhengming Wang, Xianghui Li, Qianfeng Jiang, Min Xu

**Affiliations:** Cardiovascular Medicine, The First People’s Hospital of Zunyi, Zunyi, Guizhou, China

**Keywords:** butyric acid, cardiovascular disease, hypertension, atherosclerosis, diabetes, obesity

## Abstract

Cardiovascular disease (CVD) remains a predominant cause of morbidity and mortality globally, characterized by pathological mechanisms that encompass inflammation, oxidative stress, metabolic disturbances and immune dysregulation. Recently, the influence of gut microbiota and its metabolites on the onset and progression of CVD has garnered significant attention. Short-chain fatty acids (SCFAs), particularly butyrate, are the primary products of gut microbial fermentation of dietary fiber. Butyrate is instrumental in maintaining intestinal barrier function and immune homeostasis and exhibits notable anti-inflammatory, antioxidant, and metabolic regulatory potentials in cardiovascular diseases. Nonetheless, the precise molecular mechanisms of butyrate in various cardiovascular diseases and its clinical translational value necessitate a systematic review of the literature. This study conducted a comprehensive search of databases, such as PubMed and Web of Science, to synthesize recent basic and clinical research on butyrate and cardiovascular diseases, focusing on its role in hypertension, atherosclerosis, coronary artery disease, atrial fibrillation, diabetic cardiomyopathy, and heart failure. The findings indicate that butyrate can influence cardiovascular health through multiple pathways, including the modulation of G protein-coupled receptors (GPCRs), histone deacetylases (HDACs), and peroxisome proliferator-activated receptors (PPARs). Although numerous experimental studies have corroborated the protective effects of butyrate in cardiovascular diseases, its clinical translation remains challenging owing to factors such as optimal administration route, dose optimization, and individualized treatment strategies. Future research should integrate large-scale population cohort analyses and randomized controlled trials (RCTs) to ascertain the precise efficacy of butyrate in the prevention and treatment of cardiovascular diseases and explore its potential as a novel therapeutic target.

## 1 Introduction

CVD is the leading cause of mortality and morbidity worldwide. It encompasses conditions such as atherosclerosis, hypertension, and myocardial infarction, with heart failure constituting the terminal stage of various cardiovascular diseases (1, 2). Dyslipidemia, obesity, and insulin resistance are globally acknowledged as significant risk factors for CVD and are frequently associated with alterations in intestinal barrier integrity and gut microbiota composition. With the rapid advancement of the global economy and technology, individuals' lifestyles and dietary habits have undergone significant changes. Notably, the traditional “Western diet” pattern—characterized by high fat, high processed sugar, and low fiber—exerts a substantial impact on metabolic health. Empirical studies have demonstrated that such dietary patterns can markedly alter the composition and abundance of the gut microbiota, reduce the production of short-chain fatty acids (SCFAs), and concurrently increase the secretion of lipopolysaccharide (LPS), trimethylamine N-oxide (TMAO), and proinflammatory cytokines. This, in turn, exacerbates intestinal barrier dysfunction and systemic low-grade inflammation, thereby further promoting the onset and progression of obesity, type 2 diabetes mellitus, and cardiovascular diseases. Epidemiological data corroborate this trend: between 2017 and 2018, the prevalence of obesity increased significantly to 42.4%, compared to 30.5% in 2000 (3). Concurrently, the International Diabetes Federation 2021 Global Diabetes Atlas report indicated that among individuals aged 20–79, there are already 536.6 million patients with diabetes worldwide. Predictive models suggest that in the absence of effective interventions, this number will increase to 783.2 million by 2,045 (4). The intricate interplay among obesity, diabetes, and gut microbiota not only directly exacerbates the global disease burden through systemic chronic inflammation but also significantly elevates cardiovascular disease risk via multiple pathophysiological mechanisms, emerging as a critical public health challenge that demands urgent resolution (5–7). Currently, a range of strategies and policies have been formulated to mitigate the incidence of CVD and prevent its onset. In clinical practice, pharmacological interventions are pivotal in the management and treatment of cardiovascular disease and its associated complications. Furthermore, the public has increasingly adopted the approach of reducing the risk of CVD through the intake of functional foods and dietary supplements. These functional foods and dietary supplements are frequently associated with alterations in the gut microbiota (8, 9). Therefore, maintaining gut microbiota homeostasis and augmenting beneficial microbial metabolites may be effective strategies for the primary prevention of CVD. The metabolites produced by the gut microbiota include TMAO, SCFAs, and bile acid metabolites. Notably, SCFAs, primarily derived from the fermentation of dietary fibers in the colon, such as acetic, propionic, and butyric acids, play crucial protective roles in CVD (2).

Butyric acid, a four-carbon short-chain fatty acid, is predominantly synthesized by various intestinal microbiota through glycolysis via two distinct pathways: butyryl-CoA: acetate CoA-transferase, phosphotransbutyrylase, and butyric acid kinase pathways (10, 11). Within the intestinal tract, it exists in an ionic form and is primarily absorbed via the monocarboxylic acid transporter protein 1 (MCT 1), which is expressed in the parietal and basolateral membranes of colonic epithelial cells. This absorption not only provides an energy source for intestinal epithelial cells but also modulates intestinal immune function (12, 13). Upon entering the portal vein and reaching systemic circulation, butyric acid interacts with multiple host organs, playing a crucial role in anti-inflammatory and antioxidant processes, glucose and lipid metabolism regulation, immune modulation, and enhancement of neural signaling. In comparison to other dietary fibers, butyrate, recognized as the "final effector molecule" of their function, offers several advantages, including direct absorption, rapid onset of action, safety, and a range of effects.This study aimed to provide a comprehensive review of the efficacy of butyrate treatment for CVD and its mechanism of action on CVD risk factors in humans.

## 2 Main mechanism

**Figure 1:**
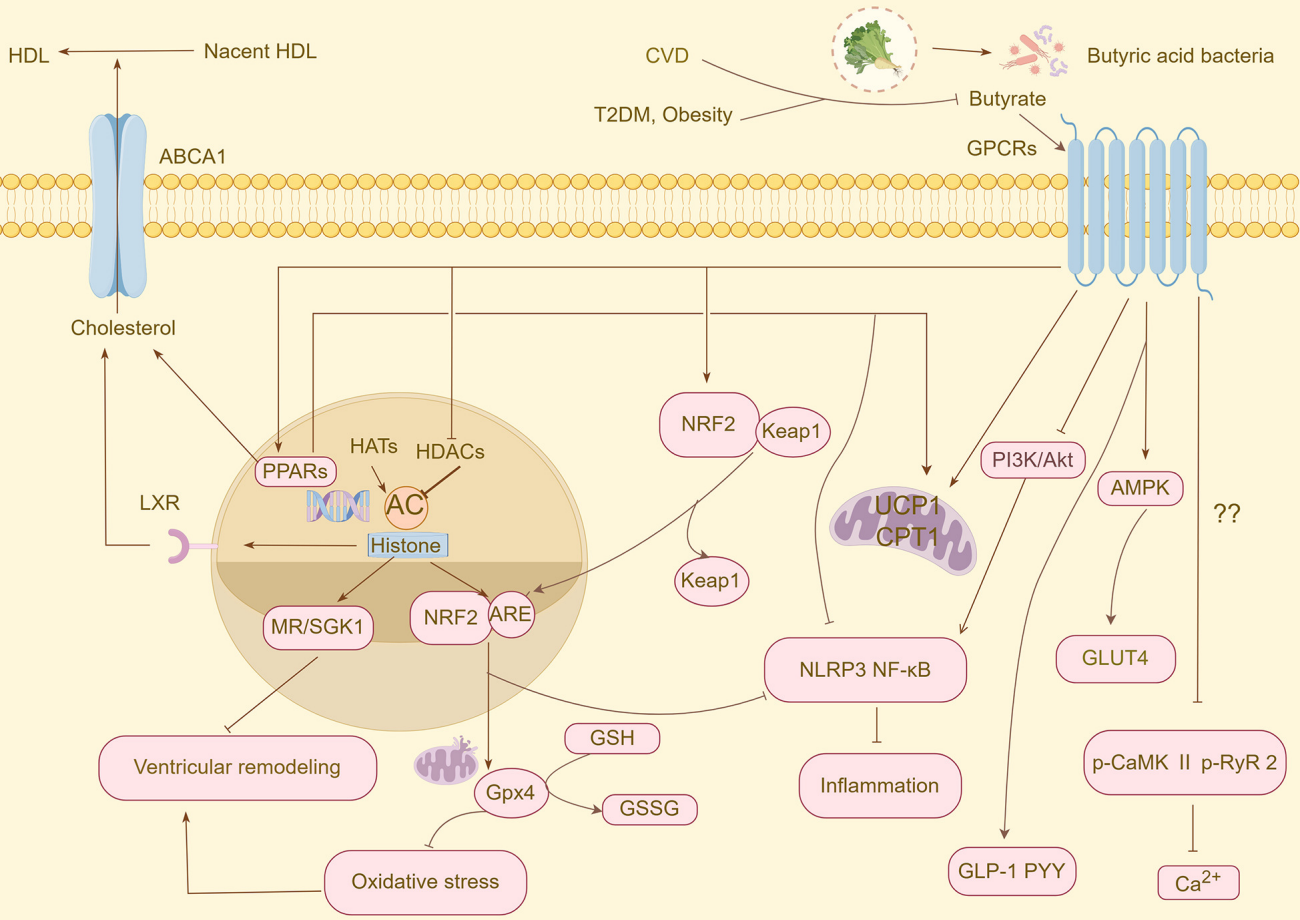
An overview of the protective mechanisms of butyrate against CVD and CVD risk factors. CVD, cardiovascular disease; T2DM, type 2 diabetes mellitus; HDL, high-density lipoprotein cholesterol; ABCA1, ATP-binding cassette transporter protein A1; GPCRs, G protein-coupled receptors; PPARs, peroxisome proliferator-activated receptors; HATs, histone acetyltransferases; HDACs, histone deacetylases; NRF2, nuclear factor erythroid 2-related factor 2; Keap 1, Kelch-like ECH-associated protein 1; ARE, antioxidant response element; GLP-1, glucagon-like peptide-1; PYY, peptide YY; MR, mineralocorticoid receptor; SGK1, glucocorticoid-dependent protein kinase 1; GSH, glutathione; GPX, glutathione peroxidase; GSSG, oxidised glutathione; AMPK, AMP-activated protein kinase; GLUT4, glucose transporter 4; PI3K, phosphatidylinositol, 3-kinase; Akt, protein kinase B; UCP1, uncoupling protein 1; CPT1, carnitine palmitoyltransferase 1; p-CaMK II, phospho-Ca_2+_/calmodulin-dependent protein kinase-II; p-RyR2, phospho-ryanodine receptor 2.

The mechanism of action of butyric acid is complex and involves multiple host pathways. Primarily, it functions as a significant epigenetic regulator by inhibiting HDACs and thereby modulating gene expression (14). In the context of epigenetic regulation, histone acetyltransferases (HATs) and HDACs establish HATs that catalyze the acetylation of lysine residues in histone tails, facilitating chromatin relaxation and gene transcription. In contrast, HDACs remove acetyl groups, resulting in chromatin condensation and gene silencing. HDACs are categorized into four classes based on their structural characteristics and cofactor dependency. Classes I (HDAC1–3, 8), II (HDAC4-7, 9–10), and IV (HDAC11) are part of the zinc-dependent Rpd3/Hda1 superfamily, whereas Class III HDACs are NAD+-dependent deacetylases. Sirtuins, belonging to Class III HDACs, can catalyze various histone (e.g., H3K9, H3K18, and H3K56) and non-histone substrates and play a pivotal role in cardiovascular diseases (15, 16). Under steady-state conditions, nuclear factor erythroid 2-related factor 2 (NRF2), a core transcription factor that regulates cellular antioxidant defense and detoxification mechanisms, predominantly resides in the cytoplasm and forms a complex with Kelch-like ECH-associated protein 1 (Keap1). Upon stimulation by NRF2 agonists, NRF2 dissociates from Keap1, undergoes nuclear translocation, and binds to the antioxidant response element (ARE). This process activates the transcription and expression of the downstream target genes. Recent studies have demonstrated that histone acetylation exerts dual regulatory effects on NRF2. On the one hand, they enhance the binding affinity between NRF2 and transcription factors in the promoter regions of target genes through epigenetic modifications. However, they inhibit the ubiquitination of NRF2, significantly reducing its proteasomal degradation rate and effectively enhancing NRF2 protein stability. This increased stability further promotes the interaction of NRF2 with members of the basic region leucine zipper family at ARE sites, ultimately strengthening the cell's defense against oxidative stress (17). Butyric acid plays a crucial role in modulating cellular responses by activating multiple receptors on the surface of the target cells. The primary receptors targeted by butyric acid are GPR43, GPR41, and Olfr78 olfactory receptors. These receptors initiate downstream signaling pathways by coupling with various G protein subtypes, such as Gq and Gi/o, thereby influencing the secretion of gastrointestinal hormones, lipolysis, inflammation, the renin-angiotensin-aldosterone system (RAAS), and the sympathetic nervous system (18). Furthermore, butyric acid functions as an endogenous PPAR agonist, exerting extensive metabolic regulatory effects through the specific activation of PPAR subtypes. Three PPAR isoforms have been identified as integral members of the nuclear receptor superfamily: PPAR*α*, PPAR*γ*, and PPARδ, each exhibiting distinct tissue distribution patterns and physiological functions. PPAR*α* is predominantly expressed in tissues with high fatty acid β-oxidation activity, such as the liver, heart, and skeletal muscle, and is involved in fatty acid uptake and oxidative metabolism by regulating the expression of target genes. In contrast, PPAR*γ* is primarily expressed in white and brown adipose tissues and acts as a key transcription factor that regulates adipocyte differentiation and lipid storage. In contrast, PPARδ is widely distributed in various metabolically active tissues. Collectively, these receptor subtypes regulate various physiological processes, including fatty acid metabolism, glucose homeostasis, discrimination, and vascular function. Activation of PPAR*α* has been shown to significantly reduce plasma triglyceride and very-low-density lipoprotein (VLDL) levels, increase high-density lipoprotein cholesterol (HDL-C) concentration, and inhibit the expression of pro-inflammatory factors. Additionally, PPAR*γ* agonists have been demonstrated to enhance insulin sensitivity and exert anti-atherosclerotic effects by regulating the cholesterol reverse transport pathway (19–21). In summary, butyrate is pivotal in numerous physiological processes, including metabolic, immune, inflammatory, and neuroendocrine regulation via various pathways (Figure 1).

## 3 Butyric acid and hypertension

Hypertension is a major public health concern worldwide. Recent research has indicated that metabolites derived from the gut microbiota can influence blood pressure regulation through various mechanisms, including vasodilation, renal sodium/potassium regulation, immunomodulation, and neurotransmitter regulation (22). Consistent findings have been reported in both animal studies and clinical trials. Notably, the abundance of butyric acid-producing bacteria in the gut microbiota is markedly reduced in animal models of simple hypertension and in hypertensive patients. Furthermore, there is a negative correlation between the abundance of butyric acid-producing flora, serum butyric acid levels, and blood pressure (23–27). In a rat model of hypertension induced by obstructive sleep apnea (OSA), Durgan et al. reported a significant reduction in the abundance of butyric acid-producing bacteria in a rat model of hypertension induced by obstructive sleep apnea and established a causal link between intestinal flora dysbiosis and the synergistic effect of OSA on hypertension through cecum transplantation (28). Gomez-Arango et al. identified a significant negative correlation between the abundance of intestinal butyrate-producing bacteria, butyrate production, and blood pressure in obese pregnant women, with similar findings reported in pregnant women with late-onset preeclampsia (29, 30). Interestingly, fecal butyrate levels were elevated in hypertensive patients compared to normotensive individuals and showed a significant correlation with 24-hour mean blood pressure, a phenomenon potentially attributable to impaired sodium-coupled monocarboxylate transporter protein 1 (SMCT 1) in the colon (29, 31). The findings of these studies are presented in the table. To elucidate the causal relationship between butyric acid and hypertension, Kim et al. administered butyric acid to C57BL6 mice infused with angiotensin II (Ang II), resulting in a significant reduction in mean arterial pressure (24).

Extensive research has been conducted on the mechanisms by which butyric acid regulates blood pressure. Current understanding suggests that GPR41 and Olfr78 play roles in blood pressure regulation, with both being expressed in smooth muscle cells of small resistance vessels. Olfr78 activation is associated with renin-dependent antihypertensive effects (32). Wang et al. demonstrated that sodium butyrate (NaB) inhibits renal (pro)renin receptors and the intrarenal renin-angiotensin system, thereby suppressing Ang II-induced hypertension (33). GPR41 knockout mice exhibit systolic hypertension, and Onyszkiewicz et al. observed that the modulation of GPR41/GPR43 by butyric acid reduced the mean arterial pressure in Wistar rats (34, 35). Furthermore, a high-salt diet is a significant contributor to hypertension, with mineralocorticoid receptor (MR) and glucocorticoid-dependent protein kinase 1 (SGK1) being crucial regulators of the salt homeostasis. The transcriptional activities of MR and SGK1 are regulated by histone acetylation. NaB alleviates high salt-induced glomerular injury and regulates water-sodium balance to improve hypertension by affecting MR transcription levels (36). In addition, endothelial nitric oxide synthase (eNOS), which is specifically expressed in the vascular endothelium, is influenced by histone deacetylation. Researchers have shown that Histone deacetylase inhibitors can induce eNOS expression in vascular smooth muscle cells and play a vital role in reducing smooth muscle cell proliferation when administered with NaB (37). The expression of inflammatory factors such as IL17, INF*γ*, and IL1β leads to vascular endothelial dysfunction and phenotypic transformation of vascular smooth muscle cells (VSMC). Butyrate is implicated in blood pressure regulation by repairing the intestinal barrier and reversing Th17 cell polarization (24, 38, 39). NOD-like receptor thermal protein domain-associated protein 3 (NLRP3) is a critical inflammatory vesicle involved in the pathogenesis of various diseases, including atherosclerosis, heart failure, and metabolic syndrome. Sun et al. found that NLRP3 activation promotes phenotypic transformation and proliferation of hypertensive VSMCs, and subsequent studies confirmed its role in hypertension through pharmacological inhibition of NLRP3 (40, 41). Several studies have shown that butyrate, a significant short-chain fatty acid, inhibits NLRP3 and exhibits potent anti-inflammatory effects (42, 43). It is widely acknowledged that blood pressure regulation is mediated by the hypothalamus-adrenal-sympathetic neural axis, with neuroinflammation being a key factor in sympathetic nerve overexcitation. Microglia release various proinflammatory cytokines, including TNF-α, IL-1β, and IL-6, which contribute to hypertension development (44). Neurological studies have demonstrated that butyrate inhibits microglial-mediated neuroinflammation (45). Reduced expression levels of the butyrate-sensitive receptors FFAR2 and FFAR3 have been observed in the paraventricular nucleus of the hypothalamus in spontaneously hypertensive rats, suggesting that the gut-brain axis may represent a novel pathway for butyrate-mediated blood pressure regulation (29). Additionally, butyrate has been shown to modulates cardiac sympathetic and vagal excitability (24, 34) (Table 1).

**Table 1:** The effect of butyrate in hypertension.

Article	Type of study	Butyrate dose	Research target	Result
Kim et al., (24)	*in vivo*	1 g/kg	C57BL6 mice infused with angiotensin II	Improved spontaneous cardiac baroreceptor reflexes and cardiac sympathetic tone
				Reduced mean arterial pressure
				Reversed the increase in Th17 cells
				Improved myocardial hypertrophy
Wang et al., (33)	*in vivo*	1 μg/kg min	SD rats with uninephrectomized and angiotensin II injection	Inhibited renal the PRR and renal angiotensin expression levels
				Reduced mean arterial pressure
	*ex vivo*	2 μmol/L	The innermedullary collecting duct cells	Regulated PRR expression and renin release
Onyszkiewicz et al., (34)	*in vivo*	0.14,1.4,2.8,5.6 mmol/kg	Wistar rats	Regulated GPR41/43 expression
	*ex vivo*	5 μM–1 mM	Mesentery and gracilis arteries	Improved arterial diastolic function
Wu et al., (36)	*in vivo*	1 g/kg	Deoxycorticosterone acetate/salt-induced hypertensive rats	Decreased renal TNF-α and IL-6 mRNA levels, and increased IL-10 mRNA levels
				Inhibited MR and SGK1 expression on renal tubules
	*ex vivo*	0.5 µM	HK2 cell line	Inhibited aldosterone-induced MR/SGK1 expression

Th17, T helper 17; PRR, (pro)renin receptor; GPR, G-protein-coupled receptor; TNF-α, tumor necrosis factor-alpha; IL-6, interleukin-6; IL-10, interleukin-10; MR, mineralocorticoid receptor; SGK1, glucocorticoid-dependent protein kinase 1; HK, human kidney.

## 4 Butyric acid and atherosclerosis

Atherosclerosis (AS), a predominant cause of cardiovascular disease, is characterized by a progressive inflammatory condition of the arterial vessel wall, facilitated by pro-inflammatory cytokines, inflammatory signaling pathways, bioactive lipids, and adhesion molecules (46, 47). An imbalance in gut microecology is a potential contributing factor to the onset of atherosclerosis, with butyric acid, a metabolite of intestinal flora, serving as a crucial short-chain fatty acid in mitigating atherosclerosis (48–51). Aguilar et al. demonstrated that dietary supplementation with butyrate diminishes atherosclerotic lesions and enhances plaque stability by inhibiting NF-κB activation (52). Butyrate decreases the levels of IL-1β, IL-6, and TNF-α in human aortic endothelial cells by upregulating intestinal tight junction proteins and restoring intestinal immunity, thereby ameliorating inflammatory damage in the arterial wall (53). Under pathological conditions, NLRP3 inflammasomes induce autocatalysis and activation of caspase-1, leading to the release of proinflammatory cytokines, which are pivotal in the progression of atherosclerosis. In a partially ligated carotid artery mouse model, butyrate significantly reduced the formation and activation of NLRP3 inflammasomes in the carotid artery wall of Asc+/+ mice, which was comparable to that observed in Asc-/- mice. In cultured EOMA cells, butyrate inhibited the formation and activation of NLRP3 inflammasomes induced by 7-ketocholesterol and cholesterol crystals (54). Previous studies have suggested that butyrate also influences the regulation of macrophage M1/M2 phenotypic transition. Butyrate stimulates hepatic ketogenesis, and the ketone body β-hydroxybutyrate may promote macrophage M2 polarization by inhibiting the NLRP3 inflammasome. However, this may be associated with the regulation of GPR43/HDAC-miRNAs (55–58). Adhesion molecules, which mediate cell-cell and cell-extracellular matrix recognition and adhesion, contribute to the development of atherosclerosis by mediating tissue inflammation, immune response, and other mechanisms (46). Butyrate significantly inhibits the expression of intercellular adhesion molecule-1, intercellular adhesion molecule, and E-selectin, thereby reducing the adhesion of human monocytes (THP-1 cells) to human aortic endothelial cells (HAECs) (53, 54, 59).

Oxidative stress induces inflammation, facilitating the development of arterial lipid plaques in atherosclerosis. Reactive oxygen species (ROS) influence the formation of oxidized low-density lipoproteins and promote various forms of cell death, including pyroptosis, apoptosis, and ferroptosis (60). Nicotinamide adenine dinucleotide oxidase (NOX) is an important source of ROS. Aguilar et al. demonstrated that oral administration of butyrate inhibited NOX activity in endothelial cells and reduced macrophage migration and activation at the lesion site, thereby mitigating oxidative stress and inflammation in the brain. This effect may be associated with the regulation of the PPARδ/miR-181 pathway (61, 62). NRF2 is a crucial transcription factor in the antioxidative stress response, primarily acting on the cystine/glutamate antiporter (System Xc-), GPX4, GSH, and heme oxygenase 1, among others. Its potent agonists have been shown to ameliorate nearly all disease-like phenotypes in cardiovascular disease models, including heart failure, myocardial infarction, cardiomyopathy, and atherosclerosis (63–65). Butyrate is a potent NRF2 agonist that modulates glutathione/glutathione S-transferase activity to scavenge ROS in VSMC (66, 67). Mathew et al. found that butyrate treatment of VSMC not only upregulated GPX4 expression but also enhanced the overall catalytic activity of GPX and inhibited the expression of inflammatory genes targeted by NF-*κ*B (68). GPX4 is pivotal in regulating ferroptosis, and its unique function of inhibiting lipid peroxidation by converting lipid hydroperoxides into nontoxic alcohols. However, the mechanism by which butyric acid inhibits ferroptosis in atherosclerosis remains unclear (69, 70). Phagocytosis of oxidized low-density lipoproteins (LDL) by macrophages and smooth muscle cells to form foam cells is a critical step in the development of atherosclerosis. Butyrate has been shown to reduces cholesterol uptake by inhibiting Niemann-Pick C1-like protein 1 (NPC1L1), an intestinal phytosterol and cholesterol transporter protein, through the upregulation of hepatic X receptor transcriptional activity (71). ATP-binding cassette transporter protein A1 (ABCA1) is an essential cholesterol transporter protein regulated by liver X receptor alpha (LXR*α*) and PPARs that facilitates cholesterol efflux for HDL synthesis and inhibits foam cell formation (72, 73). Du et al. found that butyrate protects against high-fat diet-induced atherosclerosis by mediating the activation of ABCA1 in macrophages of ApoE-deficient mice through specificity protein 1 (Sp1) (74). Additionally, histone deacetylase 3 (HDAC3) reduces ABCA1 expression by decreasing LXR*α* promoter levels, suggesting a potential role for butyrate in inhibiting LDL and foam cell formation (50) (Table 2).

**Table 2:** The effect of butyrate in atherosclerosis.

Article	Type of study	Butyrate dose	Research target	Result
Aguilar et al., (52)	*in vivo*	Feed containing 1% sodium butyrate	ApoE-/- mice	Reduced macrophage migration at lesion sites and increased collagen deposition and plaque stability
				Reduced aortic atherosclerotic lesions
	*ex vivo*	0, 0.1, 0.5, 1.0 mM	EA.Hy926 cells	Reduced oxLDL uptake
				Reduced nuclear translocation of the p65 subunit
	*ex vivo*	0.5 mM	Macrophages	Reduced oxLDL uptake and intracellular accumulation
Yuan et al., (54)	*in vivo*	500 mg/kg	Mice with partially ligated carotid arteries	Blocked the formation of NLRP3 inflammatory vesicles
	*ex vivo*	1 mM	EOMA cells	Inhibited the formation of NlRP3 inflammatory vesicles
Ma et al., (55)	*in vivo*	200 mg/kg	ApoE-/- mice	Promoted M2 polarization
				Inhibited the expression of IL-1β, IL-6, IL-17A, IFN-*γ*
				Up-regulated GPR43 expression and down-regulated HDAC 3
Shen et al., (53)	*ex vivo*	50, 100 μM	HAECs, THP-1 cells	Reduced ICAM-1 and VCAM-1 expression levels
				Reduced levels of IL-1β, IL-6 and TNF-α
Wang et al., (59)	*ex vivo*	100, 200 μM	HUVECs, THP-1 cells	Reduced IL-8 and MCP-1 secretion
				Inhibited VCAM-1 and E-selectin expression
Aguilar et al., (61)	*ex vivo*	0.5 mM	EA.Hy926 cells	Reduced expression of NOX subunit p22phox and CCL2 production
Tian et al., (62)	*in vivo*	0.5 mg/g	ApoE-/- mice and PPARδ-/-mice	Reduced production of ROS
	*ex vivo*	100 μmol/L	mBMECs	Reduced NOX2 expression
				Increased miR-181b expression
Ranganna et al., (66)	*ex vivo*	5 mM	VSMCs	Improved the overall catalytic activity of GSH⁄GST
				Reduced levels of ROS
Mathew et al., (68)	*ex vivo*	0, 1, 2, 3, 4, 5, 6, 7, 8 mM	VSMCs	Inhibited the synthesis of NF-*κ*Bp65, IKKα, IKKβ and IkBα
				improved overall GPX activity
				Increased GPX3 and GPX4 expression
Chen et al., (71)	*ex vivo*	0, 0.01, 0.1, 1 mmol/L	Caco-2	Reduced NPC1L1 and inhibited cholesterol uptake
				Enhanced LXRE-Luc transcriptional activity
Du et al., (74)	*in vivo*	200 mg/kg·day,400 mg/kg·day	ApoE-/- mice	Reduced the formation of atherosclerotic lesions
Du et al., (74)	*ex vivo*	0.5, 1, 2, 5 mM	Murine RAW 264.7 macrophages	Increased ABCA1 protein levels and cholesterol efflux
				Decreased Sp1 levels

ApoE, apolipoprotein E; oxLDL, oxidized low-density lipoprotein; NlRP3, NOD-like receptor thermal protein domain-associated protein 3; IL-1β, Interleukin-1β; IL-17A, interleukin-17A; IL-8, interleukin-8; IFN-γ, interferon-gamma; TNF-α, tumor necrosis factor-alpha; MCP-1, monocyte chemoattractant protein-1; CCL2, C-C motif chemokine ligand 2; PPARδ, peroxisome proliferator-activated receptor delta; mBMECs, mouse brain microvascular endothelial cells; VSMCs, vascular smooth muscle cells; NF-κB, nuclear factor-κB; IKK, IκB kinase; Caco, human colorectal adenocarcinoma cells; HDAC 3, histone deacetylase 3; ICAM-1, intercellular adhesion molecule 1; VCAM-1, vascular cell adhesion molecule 1; NOX, NADPH oxidase; GSH, glutathione; GST, glutathione S-transferase; ROS, reactive oxygen species; GPX, glutathione peroxidase; NPC1L1, niemann-pick C1-like protein 1; LXRE-Luc, liver X receptor response element-driven luciferase; ABCA1, ATP-binding cassette transporter protein A1; Sp1, specificity protein 1.

## 5 Butyric acid and coronary artery disease

Coronary artery disease (CAD) is a major cause of mortality worldwide. One study identified associations between the gut microbiome and metabolic alterations with the severity of CAD, noting a significant reduction in butyric acid-producing Trichoniculidae and Rumen cocci in patients with CAD compared to healthy controls. It has been posited that butyric acid-producing Faecalibacterium and Bayeriella rosans may sustain normal coronary artery physiology through their interactions with various serum metabolites (75). Liu et al. analyzed the microbial community using 16S rRNA gene sequencing and shotgun metagenomic sequencing, revealing a low molar proportion of butyrate in stool samples from patients with acute myocardial infarction (AMI) and a negative correlation between the abundance of Roseberry and AMI severity (76). Additionally, patients with acute coronary syndrome (ACS) exhibit a relative reduction in butyrate-producing bacteria, including Clostridium spp., Hadrus anaerobic rods, Streptococcus thermophilus, and Blauderia (77). Similarly, research involving young patients with AMI indicated that the gut metabolite butyric acid may exert a protective effect against coronary artery disease (78).

Butyric acid, a gut microbiota-derived metabolite, has been implicated in the repair process after myocardial infarction (MI) (79, 80). Cheng et al. developed NaB-loaded poly(lactic acid-hydroxyacetic acid)-poly(N-isopropylacrylamide) microspheres (PP-N) to extend the release of NaB, which were then injected into the myocardial ischemic region of an AMI rat model. These findings indicate that NaB released from the PPN, in conjunction with Sirt3, activates various biological functions, reduces ROS production in the infarct area, and significantly enhances the systolic and diastolic functions of the rat heart, surpassing the effects of direct NaB injection (81). Furthermore, β-hydroxybutyric acid, a butyric acid metabolite, exhibits anti-inflammatory properties in patients with AS. Through clinical studies and animal experiments, Chen et al. suggested that the cardioprotective effect of butyric acid in myocardial infarction may be associated with increased plasma β-hydroxybutyric acid levels and improved intestinal function in a dose-dependent manner (80). The high mortality rate associated with acute myocardial infarction is attributed to malignant arrhythmias and heart failure. Malignant arrhythmias in infarcted hearts are associated with excessive sympathetic innervation and inflammatory responses. Jiang et al. demonstrated that butyrate administration could reduce myocardial infarction size and prevent post-infarction ventricular arrhythmia by inhibiting cardiac sympathetic remodeling and inflammatory responses (82).

Early ischemia-reperfusion therapy can reduce the mortality risk in patients with acute myocardial infarction. However, secondary damage to cardiomyocytes, termed Myocardial Ischemia-Reperfusion Injury (MIRI), occurs during this therapy. This process involves elevated ROS levels within cardiomyocytes, disrupting the balance of myocardial oxidative and antioxidant systems, accompanied by M1 macrophage polarization, calcium overload, and other factors, ultimately leading to cellular necrosis, apoptosis, and ferroptosis (83–86). Hu et al. demonstrated that an intraperitoneal injection of 300 mg/kg NaB significantly mitigated the increase in malondialdehyde levels and decrease in superoxide dismutase levels induced by myocardial ischemia-reperfusion, thereby alleviating oxidative stress and the inflammatory response (87). Conversely, Lim et al. explored whether intraperitoneal injection of 10 mg/kg sodium acetate, sodium propionate, and NaB administered one hour prior to coronary artery ligation could positively affect myocardial ischemia-reperfusion injury. Their results indicated that NaB and sodium propionate reduced cardiac infarct size by inhibiting apoptosis, with the anti-apoptotic effects of NaB being evident in both the infarct and border zones (88). A subsequent study further elucidated the role of butyric acid in reversing MIRI-induced autonomic dysfunction by inhibiting the paraventricular nucleus and superior cervical ganglia (89) (Table 3).

**Table 3:** The effect of butyrate in coronary artery disease.

Article	Type of study	Butyrate dose	Research target	Result
Cheng et al., (81)	*in vivo*	5 μg	Rats with acute myocardial infarction	Improved systolic and diastolic function of the heart
				Inhibited the expression of the NOX subunit gp91phox
				Activated Sirt3
Jiang et al., (82)	*in vivo*	1 mol/L (butyric acid,7.5 ml/kg)	Rats with acute myocardial infarction	Reduced myocardial infarct size and improved susceptibility to malignant ventricular tachyarrhythmias
				Inhibited TNF-α and IL-1β expression and promoted IL-10 production
				Improved cardiac sympathetic remodeling
Hu et al., (87)	*in vivo*	300 mg/kg	Rats with myocardial ischemia-reperfusion injury	Inhibited TNF-α and IL-6 expression
				Reduced MDA levels and increased SOD levels
				Reduced myocardial I/R-induced infarct size
Lim, (88)	*in vivo*	10 mg/kg	Rats with myocardial ischemia-reperfusion injury	Reduced the proportion of apoptotic cells in the area of myocardial infarction and the border zone
Yu et al., (89)	*in vivo*	200 mmol/L	Rats with myocardial ischemia-reperfusion injury	Improved oxidative stress response and apoptosis
				Inhibited cFos expression in PVN and SCG

NOX, NADPH oxidase; Sirt3, sirtuin 3; TNF-α, tumor necrosis factor-alpha; IL-1β, interleukin-1β; IL-6, interleukin-6; MDA, malondialdehyde; SOD, superoxide dismutase; PVN, paraventricular; SCG, nucleus superior cervical ganglion.

## 6 Butyric acid and atrial fibrillation

Atrial fibrillation (AF) is one of the most prevalent cardiac arrhythmias and is associated with an increased risk of vascular embolic disease, mortality, and disability. Current research suggests a connection between gut dysbiosis, inflammation, and the development of AF. Zhang et al. investigated the hypothesis that gut dysbiosis promotes age-related AF through activation of the NLRP3 inflammasome induced by LPS and glucose (90). In examining the potential effects of gut microbiota-derived SCFAs on AF, some researchers have identified disruptions in genes related to SCFA synthesis and observed reduced levels of fecal SCFAs, including acetic, propionic, and butyric acids, in patients with AF. *Ex vivo* experiments have demonstrated that SCFAs may prevent AF by improving cardiac electrical remodeling and fibrosis via the GPR43/NLRP3 signaling pathway. The primary mechanism involves SCFAs preventing the overexpression of calcium/calmodulin-dependent protein kinase II(CaMKII) phosphorylation and its associated ryanodine receptor 2 (RyR2) phosphorylation in the atria, thereby alleviating calcium disruption (91). Given its potent anti-inflammatory properties, butyric acid may offer a protective effect against AF; however, further investigation is required.

## 7 Butyric acid and diabetic cardiomyopathy

In 1972, Rubler et al. identified a novel cardiomyopathy in patients with diabetes, termed diabetic cardiomyopathy (DCM). This condition is characterized by hyperglycemia-induced diastolic contractile dysfunction of the heart in the absence of coronary artery disease, hypertension, or valvular heart disease (92). DCM is characterized by cardiac fibrosis, cardiomyocyte hypertrophy, and microvascular pathology, with primary mechanisms involving impaired metabolic pathways, oxidative stress, inflammation, and cell death. Intestinal flora and its metabolites are significantly associated with diabetic cardiomyopathy (93, 94). Guo et al. conducted a study examining the diagnostic value of plasma SCFAs levels and hypoxia-inducible factor 3A (HIF 3A) intron 1 methylation in patients with DCM. The study revealed that patients with DCM exhibited lower plasma butyric acid levels and increased overall mean methylation in HIF 3A intron 1 compared with patients with diabetes mellitus (DM). The receiver operating characteristic curve indicated that the area under the curve for the combination of plasma butyric acid levels and HIF 3A intron 1 CpG 6 methylation was 0.737, with a sensitivity of 75% and specificity of 79% at a cutoff value of 0.69. This suggests that the association between plasma butyric acid and HIF 3A intron 1 CpG 6 methylation may represent a potential mechanism for DCM pathogenesis and that the combined detection of these levels may serve as a diagnostic tool for DCM (95). The addition of 1% butyrate to the drinking water of streptozotocin-induced diabetic mice demonstrated that butyrate ameliorated myocardial hypertrophy and fibrosis by inhibiting histone deacetylase activity and apoptosis, while inducing angiogenesis in the myocardium (96). Furthermore, butyrate plays a role in regulating impaired insulin metabolism signaling, which is discussed below (Table 4).

**Table 4:** The effect of butyrate in diabetic cardiomyopathy.

Article	Type of study	Butyrate dose	Research target	Result
Chen et al., (96)	*in vivo*	1% NaB drinking water	Diabetic mice	Reduced cardiomyocyte diameter and myocardial interstitial collagen deposition
				Reduced HDAC activity
				Improved apoptotic signaling

NaB, sodium butyrate; HDAC, histone deacetylase.

## 8 Butyric acid and heart failure

Heart failure (HF) is characterized by impaired systolic and diastolic functions of the heart and represents the terminal stage of nearly all cardiovascular diseases, imposing an economic burden and significantly diminishing the quality of life of patients with HF. Disruptions in cardiac energy metabolism are critical in the pathogenesis of HF, leading to an inadequate cardiac energy supply, which ultimately results in cardiac pump failure and systemic energy metabolism. Fatty acids serve as the primary carbon source for cardiomyocytes, and the fatty acid oxidation pathway depends on the carnitine palmitoyltransferase system to transport fatty acids to the mitochondria for energy production (97). The reduced activity of carnitine palmitoyltransferase 1 (CPT 1) on the outer mitochondrial membrane in failing hearts indicates impaired fatty acid oxidation in cardiomyocytes, prompting ketone bodies to become an alternative carbon source for ATP production to meet the demands of myocardial energy metabolism. Carley et al. induced cardiac hypertrophy and dysfunction in rats through transverse aortic narrowing and subsequently perfused the isolated hearts with 13C-labeled β-hydroxybutyric acid and butyrate. They observed that the oxidative utilization of butyrate exceeded that of β-hydroxybutyric acid, suggesting that butyrate may serve as a superior alternative fuel for failing heart (98). Another study confirmed that butyrate upregulates CPT1A expression to regulate fatty acid oxidative energy supply (99). In addition to regulating cardiac metabolism, butyric acid modulates cardiac hypertrophy and exerts antifibrotic effects (100). Seefeldt et al. demonstrated that butyric acid functions as a positive inotropic agent with vasodilatory effects (101). Collectively, these findings, along with the previously established antihypertensive and antiatherosclerotic effects of butyric acid, highlight its potential anti-HF properties (Table 5).

**Table 5:** The effect of butyrate in heart failure.

Article	Type of study	Butyrate dose	Research target	Result
Carley et al., (98)	*in vivo*	1 mmol/L	Rats with coarctation of the transverse aorta	Entered the TCA produces more acetyl-CoA
				Increased coupling between β-oxidation and TCA cycling activities
Zhang et al., (100)	*in vivo*	1 g/kg/d	Male SD rats for chronic infusion of Ang II	Reduced cardiomyocyte hypertrophy
				Reduced collagen fiber deposition
				Inhibited the expression of NLRP3 and IL-1β
Seefeldt et al., (101)	*in vivo*	0.033,0.165, 0.661 g/kg	Male SD rats	Increased cardiac output
				Increased left ventricular ejection fraction
		0.1,0.5,5 mM	Isolated heart	Increased left ventricular systolic blood pressure
		0.1–30 mM	Isolated coronary arteries	Reduced vascular resistance
				Dilated blood vessels

TCA, tricarboxylic acid cycle; Ang II, angiotensin II; NLRP3, NOD-like receptor thermal protein domain-associated protein 3; IL-1β, interleukin-1β.

## 9 Butyric acid and obesity, diabetes

Obesity and diabetes, which are interconnected metabolic disorders, substantially increase the risk of cardiovascular disease through their combined effects. The fundamental pathological mechanisms involve a self-perpetuating cycle of chronic, low-grade inflammation and metabolic disturbances. Excessive accumulation of visceral adipose tissue serves as an independent risk factor for cardiovascular lesions via various mechanisms. At the tissue level, caloric surplus induces abnormal hypertrophy and proliferation of adipocytes, leading to pathological expansion of white adipose tissue and a concomitant reduction in the amount of brown fat. This remodeling of adipose tissue drives changes in adipokine secretion profiles, marked by a significant increase in pro-inflammatory factors (TNF-α, IL-6, and MCP-1) and a decrease in adiponectin, which exerts protective effects while simultaneously activating the NLRP3 inflammasome to promote IL-1β secretion. At the microenvironment level, hypoxia in adipose tissue results in a reduction in regulatory T cells and polarization of macrophages towards the pro-inflammatory M1 phenotype, establishing a chronic low-grade inflammatory state. This inflammatory milieu interacts with the characteristic metabolic abnormalities of diabetes: persistent hyperglycemia activates the polyol pathway and promotes the formation of advanced glycation end products (AGEs), which activate the NF-*κ*B signaling pathway through RAGE receptors, leading to excessive ROS production and endothelial dysfunction. At the myocardial level, metabolic remodeling occurs, including increased fatty acid oxidation, impaired glucose utilization, mitochondrial dysfunction, and increased lipotoxicity. Overactivation of the RAAS and endothelin-1 systems further promotes myocardial fibrosis and vascular remodeling, ultimately culminating in various cardiovascular complications, including atherosclerosis, acute coronary syndrome, and heart failure. The synergistic mechanism involves an inflammatory microenvironment associated with obesity, which exacerbates insulin resistance, whereas the hyperglycemic state linked to diabetes further intensifies the inflammatory response, creating a positive feedback loop that exacerbates the condition. This interaction results in an exponential increase in cardiovascular risk among patients with metabolic disorders, underscoring the need for multitargeted interventions to address the pathological network (94, 102–107). A multicenter randomized controlled trial involving overweight or obese adults with type 2 diabetes mellitus demonstrated that individuals who achieved a weight loss of at least 10% within a year experienced a 21% reduction in the risk of major outcomes, such as acute myocardial infarction and stroke, and a 24% reduction in the risk of secondary outcomes, such as hospitalization for congestive heart failure (108). Effective blood glucose control can enhance the prognosis and quality of life of patients with diabetes mellitus. Research suggests that dapagliflozin can reduce the hospitalization rate for heart failure (109). Therefore, effective management of weight and blood glucose levels is crucial for improving cardiovascular outcomes.

Although numerous strategies exist for lowering blood glucose levels and reducing body weight, the outcomes of coexisting cardiovascular conditions remain poor. To enhance the understanding of the diagnosis and treatment of metabolic disorders, researchers have identified the gut microbiota as a crucial "hidden organ" within the human body that plays a significant role in regulating energy metabolism and maintaining immune homeostasis (110, 111). While exploring therapeutic interventions for diabetes, researchers have identified that butyric acid-producing microbiota can enhance glucose metabolism, although this is not universally applicable to all bacteria (112, 113). The interplay between glucotoxicity, oxidative stress, inflammation, and other mechanisms results in the progressive decline of pancreatic B-cell function, transitioning from insulin resistance to impaired insulin secretion. NaB has been shown to effectively enhance pancreatic B cell function and insulin sensitivity. Powers et al. demonstrated that NaB modulates the expression of glucagon and insulin genes (114). Conversely, another study indicated that butyric acid might influence the expression of lysine at position 18 of histone H3. Butyrylation of lysine at this position in the promoter region induces the expression of insulin genes and promotes insulin secretion in rats. However, this process adversely affects the expression of genes associated with pancreatic β-cell identity, including Pdx 1, MafA, NeuroD1, Gck, and Slc 2a2 (115).Furthermore, NaB ameliorated endoplasmic reticulum stress by inhibiting the PERK-CHOP pathway, thereby preventing histological changes and functional impairment of pancreatic islets in diabetic rats (116). Yan et al. demonstrated that NaB inhibits autophagy and NLRP3 activation by blocking the PI3K/Akt/NF-*κ*B pathway in THP-1 cells, which in turn reduces oxidative stress, inflammation, and metabolic disorders induced by glycosylation end products (117). Intraperitoneal administration of NaB (500 mg/kg/d) in juvenile DM rats indicated that NaB facilitated the proliferation of pancreatic islet B cells by modulating the p38/ERK MAPK and apoptosis pathways (118). In investigating the anti-inflammatory properties of butyric acid in diabetes, NaB was found to suppress NF-*κ*B activation and the release of inflammatory cytokines in high-glucose-treated THP-1 cells. Conversely, in lipopolysaccharide (LPS)-treated THP-1 cells, 1 mM NaB reduced TNF-α and IFN-*γ* release. However, the difference was not statistically significant in patients with type 2 diabetes with poor glycemic control compared to normal subjects, and it is important to note that the study's sample size was small, resulting in a relatively large margin of error (119). Muscle tissue, which is primarily responsible for glucose uptake, comprises type I and II skeletal muscle fibers. Type I fibers exhibit a higher oxidative capacity and greater number of mitochondria than type II fibers. Evidence indicates that NaB significantly increases the number of type I skeletal muscle fibers and ameliorates insulin resistance in mice fed a high-fat diet (HFD). This effect may be associated with epigenetic regulation of nucleosome localization. Additionally, oral administration of NaB to mice alters mitochondrial function, enhances fatty acid oxidation, and increases thermogenesis (120, 121). Intestinal gluconeogenesis (IGN) positively influences glucose and energy homeostasis by signaling glucose release from the IGN to the brain via the portal glucose sensor through the peripheral nervous system, thereby affecting glucose metabolism. Butyric acid may activate IGN gene expression to promote intestinal gluconeogenesis (122). Glucagon-like peptide-1 (GLP-1), peptide YY (PYY), and glucose-dependent insulinotropic polypeptide (GIP), which are expressed in the intestinal mucosal epithelium, are crucial for insulin secretion and maintaining glucose homeostasis. Following butyrate administration, the pattern of insulin release in high-fat diet-fed mice coincided with the release of GLP-1, PYY, and GIP, whereas other short-chain fatty acids (SCFA) did not induce intestinal hormone secretion, except for propionate-induced GIP secretion. This suggests an indirect regulation of insulin production (123, 124). Clinical data indicate that oral butyric acid administration in patients with type 2 diabetes mellitus (T2DM) also elevates serum GLP-1 levels (125). Regarding the hypoglycemic effects of butyric acid compared with those of clinically used drugs, some studies have demonstrated that NaB improves blood glucose, glycated hemoglobin (HbA1c), insulin resistance, and lipid profiles compared with metformin. Furthermore, butyric acid may act as a co-agent with metformin to enhance insulin sensitization (126, 127). In a study by de Groot et al., targeting patients with type 1 diabetes mellitus, oral butyric acid supplementation did not significantly affect innate or adaptive immunity in patients with long-term diabetes, affecting only peripheral blood immune cells. This finding does not fully reflect the overall immune response of the human body or pancreatic tissues (128). Notably, differences exist between rat, mouse, and human tissues, necessitating further clinical studies to elucidate the efficacy of NaB in reducing glucose levels in humans (Table 6).

**Table 6:** The effect of butyrate in diabetes.

Article	Type of study	Butyrate dose	Research target	Result
Wang et al., (115)	*ex vivo*	5 mM	Rat islet cells	Inhibited the expression of Pdx 1, MafA, NeuroD 1, Gck and Slc2a2
				Increased H3K18bu levels in the Ins1 and Ins2 promoter regions
	*ex vivo*	5 mM	INS-1 cells	Promoted insulin secretion
Powers et al., (114)	*ex vivo*	0.2,1, 2, 5, and 20 mM	RIN1056A and RIN1027-B2 cell line	Increased glucagon and insulin mRNA levels
Hu et al., (116)	*in vivo*	500 mg/kg	Type 2 diabetes rats	Regulated the pancreatic endoplasmic reticulum stress PERK-CHOP pathway
Yan et al., (117)	*ex vivo*	400 μmol/L	THP-1 cells	Increased the expression of SOD and decreased the expression of ROS and MDA.
				Regulated the PI3K/Akt/NF-*κ*B pathway to inhibit autophagy and NLRP3
Khan & Jena, (118)	*in vivo*	500 mg/kg	Juvenile diabetic rats	Inhibited pancreatic B cell apoptosis Modulated the p38/ERK MAPK pathway
				Maintained glucose tolerance and homeostasis
Moon & Yun, (141)	*ex vivo*	0–30 μM	THP-1 cells	Inhibited mRNA levels of IL-6 and TNF-α
				Reduced the interaction of p300 in acetylated NF-κB and TNF-α
Wibowo et al., (119)	*ex vivo*	1 mM	peripheral blood mononuclear cells	TNF-α and IFN-*γ* expression were not significantly reduced
Gao et al., (120)	*in vivo*	High-fat diet with 5% WT/WT NaB	C57BL/6 mice	Increased energy expenditure and fatty acid oxidation
				Improved insulin sensitivity
				Increased type I oxide fiber
Henagan et al., (121)	*in vivo*	High-fat diet with 5% WT/WT NaB	C57BL/6 mice	Increased the proportion of type 1 oxide fibers
				Improved mitochondrial dysfunction.
				Modulated nuclear-encoding mitochondrial genes associated with the PPAR signaling pathway
De Vadder et al., (122)	*in vivo*	Diet with 5% WT/WT NaB	Male SD rats	Increases IGN gene expression, including G6PC and PCK1
Lin et al., (124)	*in vivo*	High-fat diet with 5% WT/WT NaB	C57BL/6 mice	Increased levels of GLP-1, GIP, PYY in plasma
Roshanravan et al., (125)	Clinical trial	600 mg/day	T2DM Patients	Increased postprandial GLP-1 concentration
Khan & Jena, (127)	*in vivo*	400 mg/kg, 800 mg/kg	T2DM rats	Reduced plasma triglyceride, cholesterol, LDL, VLDL,glucose and HbA1c levels
de Groot et al., (128)	Clinical trial	4 g/day	Patients with T1DM	Does not improve innate or adaptive immunity

INS-1, Insulinoma cell line 1; Pdx 1, pancreatic and duodenal homeobox 1; MafA, V-maf musculoaponeurotic fibrosarcoma oncogene homolog A; NeuroD 1, neuronal differentiation 1; Gck, glucokinase; Slc2a2, solute carrie; family 2 member 2; H3K18bu, histone H3 lysine 18 butyrylation; Ins1, insulin 1; Ins1, insulin 2; RIN1056A, rat insulinoma1056A; RIN1027-B2, rat insulinoma 1027-B2; SOD, superoxide dismutase; ROS, reactive oxygen species; MDA, malondialdehyde; NLRP3, NOD-like receptor thermal protein domain-associated protein 3; IL-6, interleukin-6; TNF-α, tumor necrosis factor-alpha; NaB, sodium butyrate; PPAR, peroxisome proliferator-activated receptor; IGN, intestinal gluconeogenesis; G6PC, glucose-6-phosphatase catalytic subunit; PCK1, phosphoenolpyruvate carboxykinase 1; GLP-1, glucagon-like peptide-1; GIP, glucose-dependent insulinotropic polypeptide; PYY, peptide YY; LDL, low-density lipoprotein; VLDL, very low-density lipoprotein; HbA1c, hemoglobin A1c; T2DM, type 2 diabetes mellitus; T1DM, type 1 diabetes.

The effects of butyric acid on weight loss have been demonstrated in animal studies. Aguilar et al. found that NaB inhibited adipocyte hypertrophy by upregulating PPAR-*γ* in obese ApoE- mice, while reducing leptin secretion in adipose tissue and enhancing lipocalin production, thereby decreasing adipose tissue NF-*κ*B expression and mitigating inflammatory responses in obese mice (129). Compared with metformin, NaB supplementation in diabetic mice resulted in smaller adipocytes and reduced fat accumulation, significantly inhibiting NLRP3 activation and diminishing the release of downstream inflammatory factors in the testicular and subcutaneous adipose tissues (43). Zhu et al. demonstrated that NaB modulates the expression of beige-specific genes, such as Hoxa9 and Tmem 26, and enhances the expression of mitochondrial uncoupling protein 1 (UCP1) and peroxisome proliferator-activated receptor *γ*. NaB also augments the thermogenic properties of brown adipose tissue, a phenomenon partially attributed to increased sympathetic innervation of adipose tissue (130). Furthermore, NaB enhances adipose tissue metabolic function by promoting the phosphorylation of adenosine monophosphate-activated protein kinase (AMPK) and facilitating glucose transporter 4 (GLUT4) activity in adipose tissue (131). GPR43 has been identified as a potential therapeutic target for metabolic disorders, and GPR43 deficiency leads to obesity in mice. Conversely, mice overexpressing GPR43 specifically in adipose tissue exhibit a significant reduction in body weight, suggesting that GPR43 activation in adipose tissue is a potential mechanism for butyrate-induced weight loss (132). Li et al. explored the effect of butyrate on appetite and found that oral administration of butyrate suppressed appetite by inhibiting neuropeptide Y-expressing orexigenic neurons in the hypothalamus and reducing the number of FOS-positive neurons in the nucleus tractus solitarius of the brainstem and the dorsal complex of the vagus nerve (133). In contrast, Whitt et al. reported that intestinal epithelial expression of HDAC3 promotes diet-induced obesity, whereas butyrate may mitigate HDAC3 activity to prevent obesity (134). The liver plays a crucial role in coordinating metabolic changes to ensure continuous energy production and delivery to the body. Numerous studies have investigated whether butyrate enhances liver functions. A rapid influx of energy through repeated fasting and refeeding stimulates fatty acid synthesis, resulting in triglyceride accumulation and ROS production. NaB reduces the risk of non-alcoholic fatty liver disease (NAFLD) by inhibiting the mRNA levels of fatty acid synthase and increasing the mRNA levels of fatty acid oxidation-associated Cpt1a expression. However, another study found that NaB did not elevate the expression of genes involved in fatty acid β-oxidation, which might be related to the dosage of NaB used (135, 136). Additionally, insulin-inducible genes (Insig), potent inhibitors of the cleavage and maturation of sterol regulatory element-binding proteins (SREBP), which are key transcription factors in adipogenesis, were found to reduce hepatic steatosis and improve lipid profiles in mice fed a high-fat diet by activating hepatic kinase B1 and increasing Insig-1 protein levels (137). However, relatively few studies have examined the effects of butyrate on weight control in humans with obesity. Data from a cross-sectional study indicated a negative correlation between plasma butyrate levels and body mass index (BMI) (138). Fecal microbiota transplantation can increase butyrate-producing bacteria in the gut of obese patients with T2DM, and when combined with lifestyle interventions, it can lead to more favorable changes in the recipients' microbiota and improved lipid profiles (139). In obese children, butyrate supplementation as an adjunct to standard treatment resulted in reductions in BMI, waist circumference, and insulin levels after six months (140). However, there is a paucity of research on obese populations, necessitating further investigation (Table 7).

**Table 7:** The effect of butyrate in obesity.

Article	Type of study	Butyrate dose	Research target	Result
Aguilar et al., (129)	*in vivo*	Diet with 1% NaB	Diet-induced obese ApoE-/- mice	Inhibited fat cell hypertrophy
				Reduced leptin and increased adiponectin
				Increased PPAR-γ expression and inhibited NF-κB expression
Wang et al., (43)	*in vivo*	1.0 g/kg	db/db mice	Reduced the expression of IL-1, IL-6 and TNF-α
				Reduced protein levels of NLRP3 and IL-1β
Zhu et al., (130)	*in vivo*	High-fat diet with 0.4% WT/WT NaB	C57BL/6 mice	Increased mRNA expression of beige-selective genes such as Hoxa9 and Tmem26
				Increased UCP-1 and PGC-1α protein levels
				Enhanced the activity of the adipose sympathetic nerve
Gao et al., (131)	*in vivo*	400 mg/kg	High-fat diet C57BL/6 mice	Increased the levels of p-AMPK, GLUT4, and HDAC in liver and adipose tissue
Li et al., (133)	*in vivo*	High-fat diet with 5% WT/WT NaB	APOE*3-Leiden.CETP mice	Reduced the number of FOS-positive neurons and neuronal ratios of neuropeptide Y and c-FOS within the arcuate nucleus of the hypothalamus
				Reduced the number of FOS-positive neurons in the brainstem nucleus solitary tract and dorsal vagus complex
Whitt et al., (134)	*in vivo*	80 mg/d	HDAC3ΔIEC, HDAC3ΔIEC-IND mice	Reduced HDAC enzyme activity in IECs
				Reduced body weight
Honma et al., (135)	*in vivo*	A diet high in sucrose with 5% NaB	SD male rats	Inhibited mRNA levels of Fas
				Increased the mRNA level of Cpt1a
Hattori et al., (136)	*in vivo*	A high-sucrose diet supplemented with 1% and 3% NaB	Wistar rats	Does not improve CPT1 gene expression
Zhao et al., (137)	*in vivo*	200 mg/kg	High-fat diet mice	Increased hepatic protein levels of Insig-1
				Activated LKB1
Coppola et al., (140)	Randomized, quadruple-blind, parallel-group, placebo-controlled trial	20 mg/kg body weight per day, up to a maximum of 800 mg/d	Obese children	Reduced BMI, waist circumference, and plasma insulin levels in obese children

NaB, sodium butyrate; ApoE, apolipoprotein E; PPAR-γ, peroxisome proliferator-activated receptor gamma; NF-κB, nuclear factor-κB; IL-1, interleukin-1; IL-6, interleukin-6; TNF-α, tumor necrosis factor-alpha; NLRP3, NOD-like receptor thermal protein domain-associated protein 3; Hoxa9, homeobox A9; Tmem26, transmembrane protein 26; UCP-1, uncoupling protein 1; PGC-1α, peroxisome proliferator-activated receptor gamma coactivator 1-alpha; AMPK, AMP-activated protein kinase; HDAC, histone acetylation.

## 10 Conclusions and prospects

**Figure 2:**
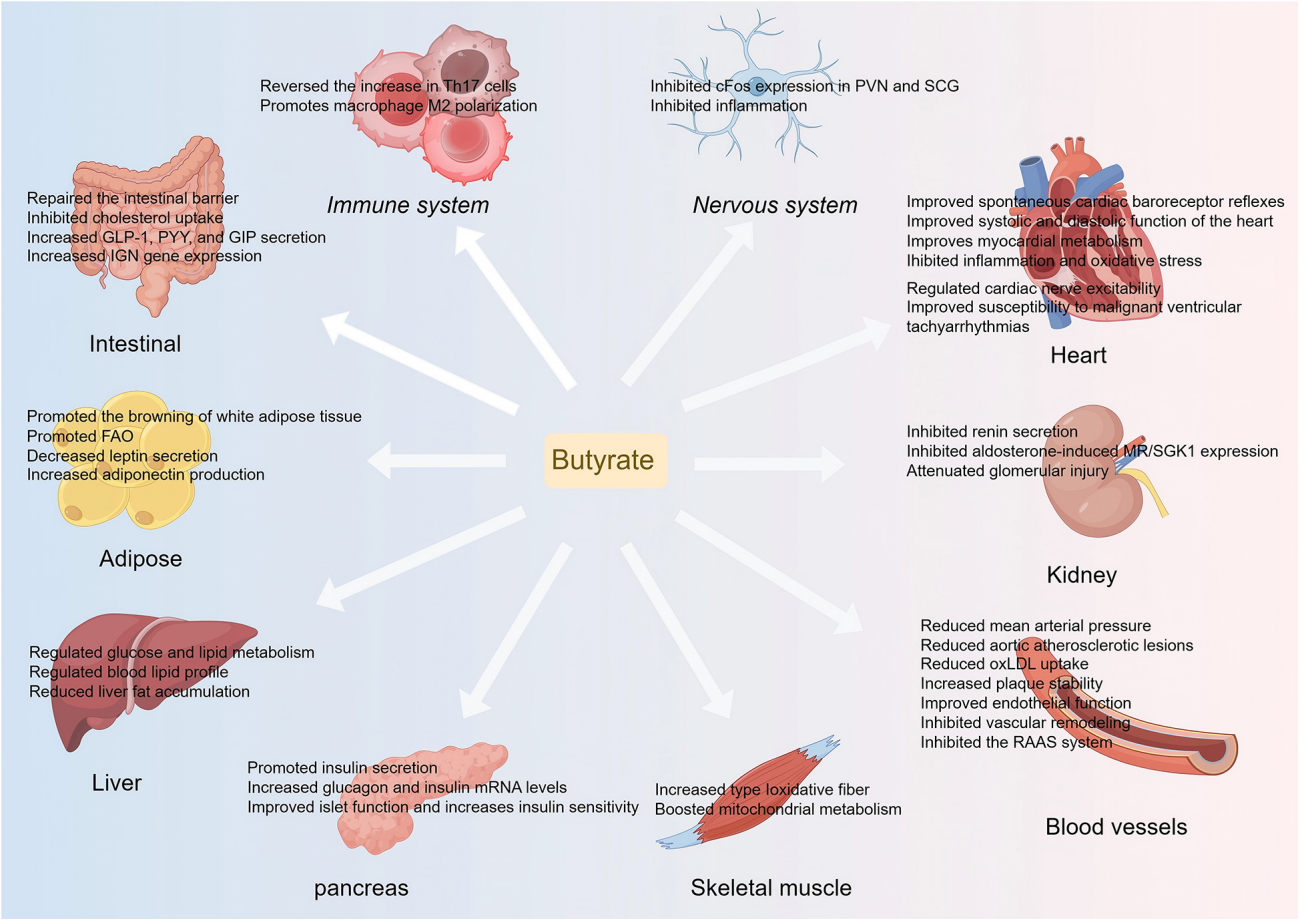
An overview of butyrate's protective effects in CVD and CVD risk factors. Th 17, T helper 17; PVN, paraventricular; SCG, nucleus superior cervical ganglion; GLP-1, glucagon-like peptide-1; GIP, glucose-dependent insulinotropic polypeptide; PYY, peptide YY; IGN, intestinal gluconeogenesis; oxLDL, oxidized low-density lipoprotein; FAO, fatty acid oxidation; MR, mineralocorticoid receptor; SGK1, glucocorticoid-dependent protein kinase 1; RAAS, renin-angiotensin-aldosterone system.

Butyrate plays a significant role in the prevention and treatment of CVD through various pathways, including anti-inflammatory, antioxidant, and metabolic regulation (Figure 2). However, current research remains predominantly in the basic stage, necessitating advancements in clinical translational research. Future research should investigate the effects of butyrate on CVD in depth from the perspectives of clinical validation, intervention optimization, mechanistic exploration, and safety assessment. Initially, large-scale RCTs are required to evaluate the differences in the efficacy of butyrate in diverse CVD populations, such as patients with hypertension and atherosclerosis. Moreover, intervention strategies should be optimized by comparing different supplementation methods (butyrate salts, probiotics, or dietary fibers) and determining optimal dosage and safety. Additionally, it is essential to assess the interactions between butyrate and cardiovascular drugs such as statins and antihypertensive medications to avoid adverse reactions. Ultimately, integrating multi-omics technologies to elucidate the regulatory mechanisms of the "butyrate-gut microbiota-cardiovascular" axis will provide a foundation for precise interventions. This framework will facilitate the translation of butyrate from basic research to clinical application, supporting the precise prevention and treatment of CVD.
